# Association between three VEGF polymorphisms and renal cell carcinoma susceptibility: a meta-analysis

**DOI:** 10.18632/oncotarget.17833

**Published:** 2017-05-12

**Authors:** Qi Hou, Mao-Yin Li, Wen-Tao Huang, Fang-Fang Wei, Jun-Ping Peng, Ming-Wu Lou, Jian-Guang Qiu

**Affiliations:** ^1^ Post-Doctoral Research Center, Department of Radiology, Longgang Central Hospital, Shenzhen Clinical Medical Institute, Guangzhou University of Chinese Medicine, Shenzhen 518116, China; ^2^ Department of Urology, Third Affiliated Hospital of Sun Yat-Sen University, Guangzhou 510630, China; ^3^ Department of Infectious Disease, Guangdong Second Provincial General Hospital, Guangzhou 510317, China

**Keywords:** vascular endothelial growth factor, renal cell carcinoma, polymorphism, meta-analysis

## Abstract

Several studies have reported an association between vascular endothelial growth factor (VEGF) gene polymorphisms rs2010963, rs3025039 and rs699947 and renal cell carcinoma (RCC). However, the results remain inconclusive and controversial. We therefore conducted a meta-analysis to evaluate this association. Electronic databases were searched for relevant case-control studies up to November 2016. RevMan 5.2 software and STATA version 12.0 were used for statistical analysis in our meta-analysis. Heterogeneity was assessed using the I^2^ value. Nine eligible studies were retrieved for detailed evaluation. The pooled estimates indicated that the GG genotype of VEGF rs2010963 polymorphism significantly decreased RCC risk [GG vs. GC+CC; GG vs. GC]. There was also a significant association between VEGF rs3025039 polymorphism and RCC susceptibility [CC+CT vs. TT; CC vs. TT]. Furthermore, a significant association between VEGF rs699947 polymorphism and RCC susceptibility was detected [A vs. C; AA+AC vs. CC; AA vs. AC+CC; AA vs. CC; AA vs. AC; AC vs. CC]. Subgroup analysis revealed that these associations held true especially for Asians. Our meta-analysis suggested that there may be a relationship between the VEGF rs2010963, rs3025039 and rs699947 polymorphisms and RCC susceptibility.

## INTRODUCTION

Renal cell carcinoma (RCC) has around 350,000 newly diagnosed cases every year and is one of the most common histologic types of kidney cancer. At the same time, the mortality of RCC is more than 140,000 per year worldwide [1, 2]. However, to date, the definite etiology of RCC is still not well defined. Several epidemiologic studies showed that the etiology of RCC is a complex interaction between environmental and multigenetic factors. Tobacco exposure [3], obesity [4] and hypertension [5] are accepted major risk factors for RCC. Recent molecular studies have reported that abnormal expression of VEGF contribute to tumorigenesis [6].

The VEGF gene is located on chromosome 6p21.3 and consists of 8 exons [7]. It plays a critical role in physiological and pathological angiogenesis which is a relatively early event in tumor development, progression and metastasis [8, 9]. Previous studies observed that functional single-nucleotide polymorphisms (SNPs) in either the regulatory or coding sequence of VEGF affects gene expression in various diseases including lung cancer [10], cardiovascular disease [11] and gastric cancer [12]. Some of VEGF SNPs, such as rs2010963 polymorphism (+405G/C, also named as -634G/C) in the 5’-untranslated region, rs3025039 polymorphism (+936C/T) in the 3’-UTR of the gene and rs699947 polymorphism (−2578C/A) in the promoter region have been suspected to correlate with RCC risk [13–17].

Recently, a series of case-control studies focusing on the associations between gene mutations (particularly SNPs) and the risk of RCC have been extensively studied [18–21]. However the results have been inconsistent. We conducted the present systematic review and meta-analysis to better understand the effects of VEGF rs2010963, rs3025039 and rs699947 polymorphisms on RCC susceptibility.

## RESULTS

### Study characteristics

**Figure 1:**
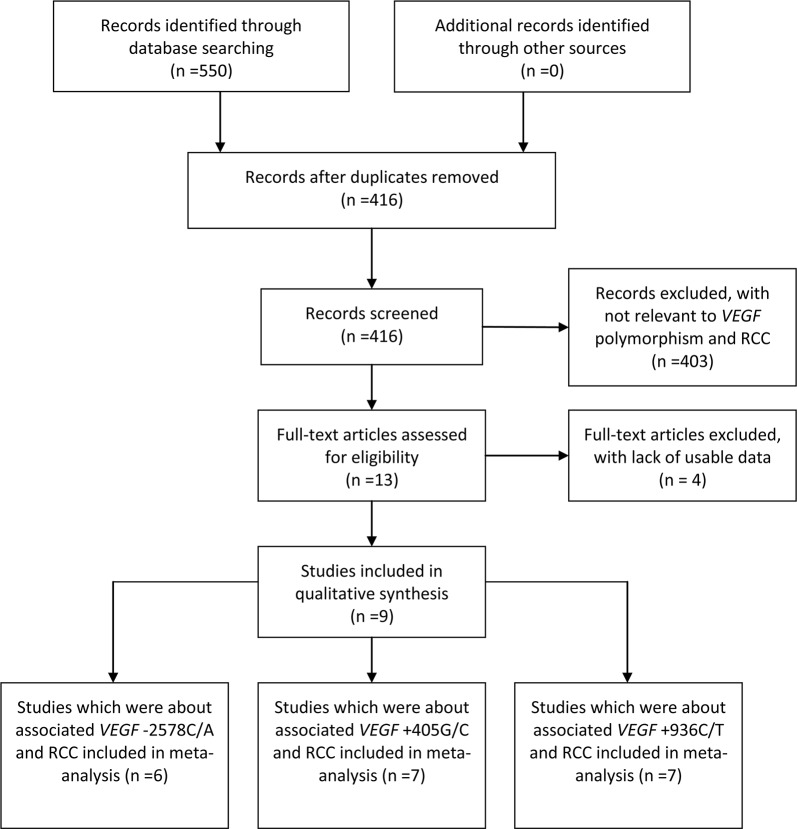
Flow diagram of study identification

A total of 550 articles were identified through a literature search in PubMed, ISI, Wangfang, Google Scholar and CNKI databases. Nine eligible studies were retrieved for detailed evaluation (Figure 1). The quality assessment was conducted using the Newcastle-Ottawa Scale (NOS) and all of studies got 6-7 points (Supplementary Table 1). We included seven studies that described an association between rs2010963 polymorphism and RCC susceptibility including 2315 cases and 3552 controls. Seven studies described an association between rs3025039 polymorphism and RCC susceptibility, including 1636 cases and 2711 controls. For rs699947 polymorphism, six studies were studied including a total of 1588 cases and 2470 controls. The genotypes of seven studies were detected by PCR-RFLP (restriction fragment length polymorphism) and two by TaqMan assay (Table 1).

**Table 1:** Characteristics of studies on VEGF polymorphisms and RCC risk included in the meta-analysis

First author	Year	Population	Genotype method	Case	Control	Genotype distribution						P HWE
						Case			Control			
rs2010963						GG	GC	CC	GG	GC	CC	
Bruyère	2010	France	PCR-RFLP	48	198	15	25	8	86	92	20	0.522
Sáenz-López	2013	Spain	TaqMan	214	279	101	93	20	129	118	32	0.528
Chao	2014	China	PCR-RFLP	824	983	287	391	146	410	429	144	0.068
Lu	2015	China	PCR-RFLP	412	824	139	194	79	299	377	148	0.127
Xian	2015	China	PCR-RFLP	266	532	30	132	104	49	256	227	0.053
Shen	2015	China	PCR-RFLP	360	360	121	170	69	134	163	63	0.273
Yang	2015	Taiwan	TaqMan	191	376	62	90	39	136	173	67	0.355
rs3025039						CC	CT	TT	CC	CT	TT	
Abe	2002	Japanese	PCR-RFLP	145	145	97	41	7	90	52	3	0.146
Bruyère	2010	France	PCR-RFLP	47	196	29	17	1	141	53	2	0.218
Sáenz-López	2013	Spain	TaqMan	215	280	156	57	2	200	73	7	0.912
Lu	2015	China	PCR-RFLP	412	824	262	91	59	554	166	104	<0.01*
Xian	2015	China	PCR-RFLP	266	532	70	127	69	196	236	100	0.056
Shen	2015	China	PCR-RFLP	360	359	224	81	55	240	73	46	<0.01*
Yang	2015	Taiwan	TaqMan	191	375	122	59	10	232	121	22	0.247
rs699947						CC	CA	AA	CC	CA	AA	
Ajaz	2011	Pakistan	PCR-RFLP	143	106	30	81	32	44	41	21	0.053
Sáenz-López	2013	Spain	TaqMan	216	272	54	114	48	77	142	53	0.388
Lu	2015	China	PCR-RFLP	412	824	171	174	67	397	332	95	0.047*
Xian	2015	China	PCR-RFLP	266	532	99	119	48	243	225	64	0.29
Shen	2015	China	PCR-RFLP	360	360	150	149	61	178	141	41	0.11
Yang	2015	Taiwan	TaqMan	191	376	106	75	10	200	153	23	0.377

* represent the genotype distribution of control sample was not meeting Hardy-Weinberg equilibrium.

### Association between rs2010963 polymorphism and RCC risk

**Figure 2:**
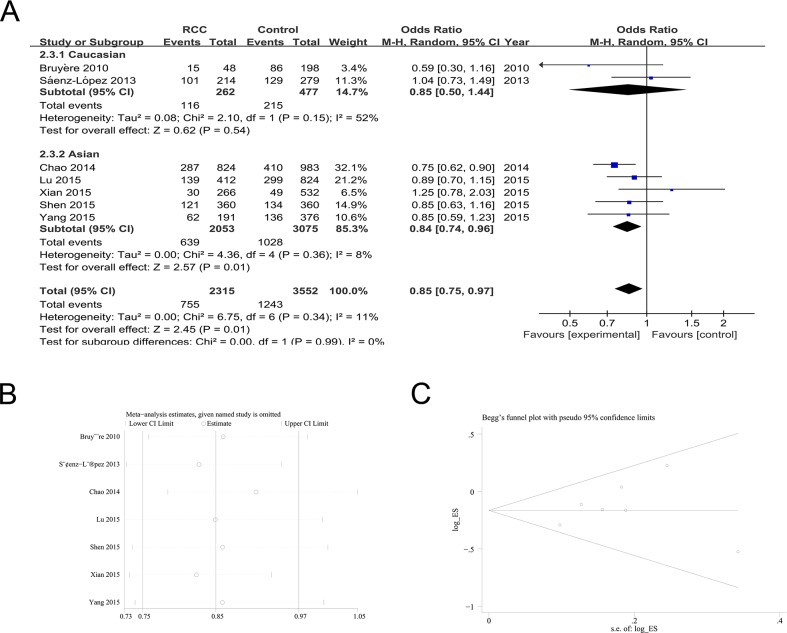
The association of rs2010963 polymorphism with RCC susceptibility in the GG vs. GC+CC model **(A)** ORs and 95%CIs. **(B)** Sensitivity analysis. **(C)** Publication bias.

The data of seven studies was available to investigate the association between rs2010963 poly-morphisms and RCC risk. The meta-analysis found a significant association between rs2010963 polymorphism and RCC susceptibility [GG vs. GC+CC (OR = 0.85; 95%CI = 0.75-0.97; *P* = 0.01; I^2^ = 11%); GG vs. GC (OR = 0.86; 95%CI = 0.76-0.97; *P* =0.01; I^2^ = 0%)] (Table 2; Figure 2A). Subgroup analysis found a similar decreased risk for RCC in the Asian population with a GG genotype [GG vs. GC+CC (OR = 0.84; 95%CI = 0.74-0.96; *P* = 0.01; I^2^ = 8%); GG vs. GC (OR = 0.85; 95%CI = 0.75-0.97; *P* =0.02; I^2^ = 0%)] (Table 2; Figure 2A).

**Table 2:** Summary ORs and 95%CI of VEGF polymorphisms and RCC risk

rs2010963	N	G vs. C			GG vs. CC			GG vs. GC			GC vs. CC			GG+GC vs. CC			GG vs. GC+CC		
		OR and 95%CI	P	I^2^%	OR and 95%CI	P	I^2^%	OR and 95%CI	P	I^2^%	OR and 95%CI	P	I^2^%	OR and 95%CI	P	I^2^%	OR and 95%CI	P	I^2^%
Total	7	0.92 (0.82, 1.02)	0.10	39	0.85(0.69, 1.04)	0.11	30	0.86(0.76,0.97)	0.01	0	0.97(0.84,1.12)	0.72	0	0.92(0.80,1.06)	0.24	0	0.85(0.75,0.97)	0.01	11
Caucasian	2	0.88 (0.56, 1.39)	0.58	67	0.80(0.29, 2.22)	0.66	68	0.89(0.61, 1.29)	0.53	13	1.02(0.58, 1.82)	0.93	15	1.00(0.61, 1.64)	0.99	54	0.85(0.50, 1.44)	0.54	52
Asian	5	0.91(0.82, 1.02)	0.09	39	0.83(0.68, 1.01)	0.07	23	0.85(0.75, 0.97)	0.02	0	0.97(0.83, 1.12)	0.67	0	0.91(0.79, 1.05)	0.22	0	0.84(0.74, 0.96)	0.01	8
rs3025039		C vs. T			CC vs. TT			CC vs. CT			CT vs. TT			CC+CT vs. TT			CC vs. CT+TT		
Total	7	0.88 (0.76, 1.01)	0.06	39	0.75(0.57, 0.99)	0.04	25	0.90(0.76, 1.06)	0.21	24	0.89(0.71, 1.11)	0.29	0	0.79(0.65, 0.97)	0.02	0	0.87(0.73, 1.04)	0.13	40
Caucasian	2	0.91 (0.56, 1.48)	0.71	53	1.33(0.22, 8.19)	0.76	40	0.87(0.58, 1.30)	0.50	18	1.77(0.46, 6.81)	0.41	0	1.46(0.28, 7.64)	0.66	30	0.88(0.54, 1.43)	0.60	42
Asian	5	0.86(0.74, 1.00)	0.05	43	0.73(0.56, 0.94)	0.02	22	0.91(0.73, 1.12)	0.36	40	0.87(0.69, 1.09)	0.22	0	0.78(0.64, 0.96)	0.02	0	0.87(0.70, 1.07)	0.18	51
HWE-yes	5	0.91(0.72, 1.15)	0.43	59	0.76(0.42, 1.37)	0.36	41	0.92(0.70, 1.21)	0.55	48	0.83(0.58, 1.19)	0.31	5	0.79(0.51, 1.22)	0.28	20	0.90(0.67, 1.21)	0.47	59
HWE-no	2	0.85(0.73, 0.99)	0.03	0	0.81(0.62, 1.07)	0.13	0	0.87(0.69, 1.09)	0.22	0	0.95(0.69, 1.31)	0.76	0	0.84(0.65, 1.10)	0.21	0	0.84(0.69, 1.02)	0.07	0
Asian HWE-yes	3	0.91(0.66, 1.25)	0.55	71	0.66(0.37, 1.15)	0.14	41	0.97(0.64, 1.47)	0.88	69	0.79(0.56, 1.09)	0.15	0	0.71(0.50, 1.00)	0.05	6	0.92(0.59, 1.43)	0.71	75
rs699947		A vs. C			AA vs. CC			AA vs. AC			AC vs. CC			AA+AC vs. CC			AA vs. AC+CC		
Total	6	1.25(1.11, 1.42)	<0.01	38	1.61(1.32, 1.97)	<0.01	0	1.23(1.02, 1.50)	0.03	0	1.27(1.03, 1.57)	0.03	52	1.33(1.09, 1.63)	<0.01	55	1.39(1.16, 1.67)	<0.01	0
Caucasian	2	1.31(0.94, 1.84)	0.12	58	1.60(0.95, 2.71)	0.08	31	1.00(0.68, 1.46)	0.99	0	1.78(0.72, 4.41)	0.21	84	1.74(0.78, 3.85)	0.17	81	1.18(0.82, 1.68)	0.38	0
Asian	4	1.24(1.07, 1.44)	<0.01	47	1.61(1.26, 2.06)	<0.01	12	1.33(1.06, 1.68)	0.01	0	1.18(1.02, 1.38)	0.03	0	1.26(1.06, 1.50)	<0.01	30	1.48(1.20, 1.83)	<0.01	0
HWE-yes	5	1.24(1.06, 1.46)	<0.01	50	1.58(1.21, 2.07)	<0.01	18	1.19(0.95, 1.50)	0.14	0	1.30(0.98, 1.72)	0.07	62	1.35(1.03, 1.77)	0.03	64	1.36(1.09, 1.69)	<0.01	0
Asian HWE-yes	3	1.21(0.96, 1.52)	0.10	64	1.54(1.04, 2.30)	0.03	41	1.33(0.99, 1.78)	0.06	0	1.16(0.95, 1.42)	0.14	9	1.23(0.94, 1.59)	0.13	52	1.46(1.08, 1.97)	0.01	13
Total	6	1.25(1.11, 1.42)	<0.01	38	1.61(1.32, 1.97)	<0.01	0	1.23(1.02, 1.50)	0.03	0	1.27(1.03, 1.57)	0.03	52	1.33(1.09, 1.63)	<0.01	55	1.39(1.16, 1.67)	<0.01	0

Sensitivity analyses revealed that the results of the pooled ORs were stable (Figure 2B for the dominant model). No evidence of publication bias was observed in the Begg's rank correlation method and Egger's weighted regression method [G vs. C (Begg's test: *P* = 1.000; Egger's test: *P* = 0.723); GG+GC vs. CC (Begg's test: *P* = 0.764; Egger's test: *P* = 0.888); GG vs. GC+CC (Begg's test: *P* = 0.764; Egger's test: *P* = 0.437); GG vs. CC (Begg's test: *P* = 0.548; Egger's test: *P* = 0.444); GG vs. GC (Begg's test: *P* = 0.548; Egger's test: *P* = 0.417);GC vs. CC (Begg's test: *P* = 0.548; Egger's test: *P* = 0.825)] (Figure 2C for the dominant model).

### Association between rs3025039 polymorphism and RCC risk

**Figure 3:**
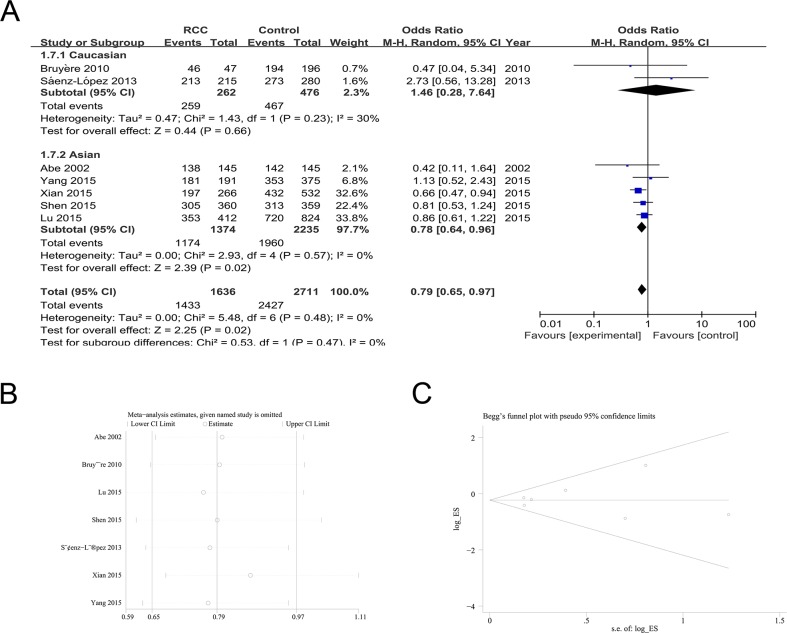
The association of rs3025039 polymorphism with RCC susceptibility in the CC+CT vs. TT model **(A)** ORs and 95%CIs. **(B)** Sensitivity analysis. **(C)** Publication bias.

Seven studies were available to investigate the association between rs3025039 polymorphisms and RCC risk. The meta-analysis found a significant association between rs3025039 polymorphism and RCC susceptibility [CC+CT vs. TT (OR = 0.79; 95%CI = 0.65-0.97; *P* = 0.02; I^2^ = 0%); CC vs. TT (OR = 0.75; 95%CI = 0.57-0.99; *P* =0.04; I^2^ = 25%)] (Table 2; Figure 3A). Subgroup analysis found a similar decreased risk for the Asian population with a C allele [C vs. T (OR = 0.86; 95%CI = 0.74-1.00; *P* =0.05; I^2^ = 43%); CC+CT vs. TT (OR = 0.78; 95%CI = 0.64-0.96; *P* =0.02; I^2^ = 0%); CC vs. TT (OR = 0.73; 95%CI = 0.56-0.94; *P* =0.02; I^2^ = 22%)] (Table 2; Figure 3A). Because the genotype distribution of control samples reported by Lu et al. and Shen et al. did not meet the Hardy-Weinberg equilibrium, we conducted the subgroup analysis excluding them. The subgroup analysis detected an association between the C allele of rs3025039 polymorphism and RCC susceptibility in the Asian population [CC+CT vs. TT (OR = 0.71; 95%CI = 0.50-1.00; *P* =0.05; I^2^ = 6%)] (Table 2).

Sensitivity analyses revealed that the results of the pooled ORs were stable (Figure 3B for the recessive model). No evidence of publication bias was observed in the Begg's rank correlation method and Egger's weighted regression method [C vs. T (Begg's test: *P* = 1.000; Egger's test: *P* = 0.409); CC+CT vs. TT (Begg's test: *P* = 0.764; Egger's test: *P* = 0.674); CC vs. CT+TT (Begg's test: *P* = 0.764; Egger's test: *P* = 0.727); CC vs. TT (Begg's test: *P* = 0.764; Egger's test: *P* = 0.673); CC vs. CT (Begg's test: *P* = 0.548; Egger's test: *P* = 0.793); CT vs. TT (Begg's test: *P* = 0.764; Egger's test: *P* = 0.874)] (Figure 3C for the recessive model).

### Association between rs699947 polymorphism and RCC risk

**Figure 4:**
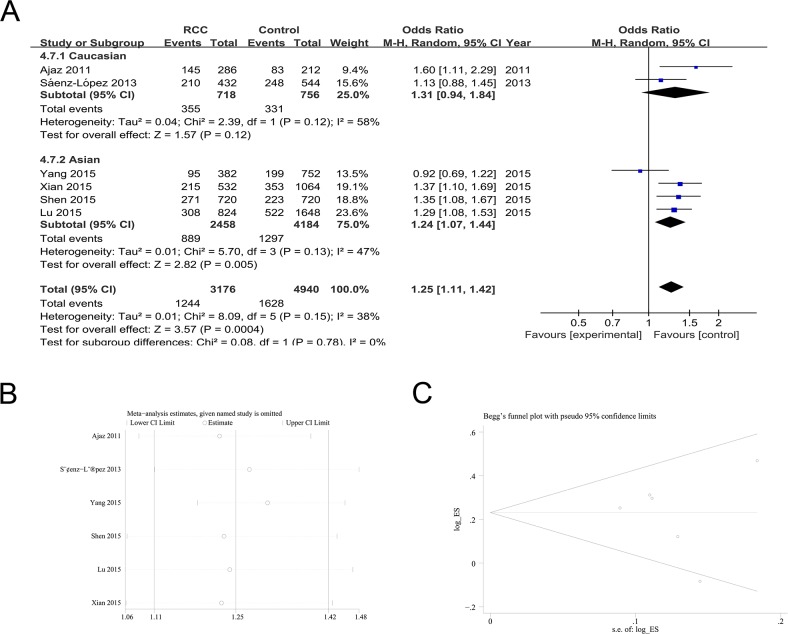
The association of rs699947 polymorphism with RCC susceptibility in the A allele vs. C allele model **(A)** ORs and 95%CIs. **(B)** Sensitivity analysis. **(C)** Publication bias.

Six studies were available to investigate the association between rs699947 polymorphisms and RCC risk. The meta-analysis found a significant association between rs699947 polymorphism and RCC susceptibility [A vs. C (OR = 1.25; 95%CI = 1.11-1.42; *P* = 0.0004; I^2^ = 38%); AA+AC vs. CC (OR = 1.33; 95%CI = 1.09-1.63; *P* = 0.006; I^2^ = 55%); AA vs. AC+CC (OR = 1.39; 95%CI = 1.16-1.67; *P* = 0.0004; I^2^ = 0%); AA vs. CC (OR = 1.61; 95%CI = 1.32-1.97; *P* <0.00001; I^2^ = 0%); AA vs. AC (OR = 1.23; 95%CI = 1.02-1.50; *P* =0.03; I^2^ = 0%);AC vs. CC (OR = 1.27; 95%CI = 1.23-1.99; *P* = 0.03; I^2^ = 52%)] (Table 2; Figure 4A).

Subgroup analysis found a similar increased risk in the Asian population with an A allele [A vs. C (OR = 1.24; 95%CI = 1.07-1.44; *P*<0.01; I^2^ = 47%); AA+AC vs. CC (OR = 1.26; 95%CI = 1.06-1.50; *P*<0.01; I^2^ = 30%); AA vs. AC+CC (OR = 1.48; 95%CI = 1.20-1.83; *P*<0.01; I^2^ = 0%); AA vs. CC (OR = 1.61; 95%CI = 1.26-2.06; *P*<0.00001; I^2^ = 0%); AA vs. AC (OR = 1.33; 95%CI = 1.06-1.68; *P* =0.01; I^2^ = 0%);AC vs. CC (OR = 1.18; 95%CI = 1.02-1.38; *P* = 0.03; I^2^ = 0%)] (Table 2; Figure 4A). Because the genotype distribution of control samples reported by Lu et al. did not meet the Hardy-Weinberg equilibrium, we conducted the subgroup analysis excluding it. This subgroup analysis detected a significant association between rs699947 polymorphism and RCC [A vs. C (OR = 1.24; 95%CI = 1.06-1.46; *P*<0.01; I^2^ = 50%); AA+AC vs. CC (OR = 1.35; 95%CI = 1.03-1.77; *P*=0.03; I^2^ = 64%); AA vs. AC+CC (OR = 1.36; 95%CI = 1.09-1.69; *P*<0.01; I^2^ = 0%); AA vs. CC (OR = 1.58; 95%CI = 1.21-2.07; *P*<0.01; I^2^ = 18%)], even in the Asian population [AA vs. AC+CC (OR = 1.46; 95%CI = 1.08-1.97; *P*=0.01; I^2^ = 13%); AA vs. CC (OR = 1.54; 95%CI = 1.04-2.30; *P*=0.03; I^2^ = 41%)] (Table 2).

Sensitivity analyses revealed that the results of the pooled ORs were stable (Figure 4B for the allelic model). No evidence of publication bias was observed in the Begg's rank correlation method and Egger's weighted regression method [A vs. C (Begg's test: *P* = 1.000; Egger's test: *P* = 0.825); AA+AC vs. CC (Begg's test: *P* = 0.707; Egger's test: *P* = 0.469); AA vs. AC+CC (Begg's test: *P* = 0.133; Egger's test: *P* = 0.057); AA vs. CC (Begg's test: *P* = 0.707; Egger's test: *P* = 0.454); AA vs. AC (Begg's test: *P* = 0.133; Egger's test: *P* = 0.040);AC vs. CC (Begg's test: *P* = 0.707; Egger's test: *P* = 0.254)] (Figure 4C for the allelic model).

## DISCUSSION

Angiogenesis, the generation of new capillary from basal vessels under both physiological and pathological conditions, is a relatively early event in tumor growth and metastasis [8, 22]. VEGF, a common pro-angiogenic growth factor, is associated with angiogenesis in multiple kinds of tumor including breast [23], colorectal [24] and kidney cancer [6]. A previous meta-analysis conducted by Gong et al. reported that VEGF gene rs699947 polymorphism and rs3025039 polymorphism correlates with an increased susceptibility of RCC [25]. However, this meta-analysis mistakenly treated VEGF gene SNP +405G/C and -634G/C, two aliases of rs2010963, as different polymorphisms. Furthermore, this study did not reveal the influence and heterogeneity from the including studies which were diverse in ethnicity. Another study by Yang et al stated that there was no significant association between VEGF gene polymorphisms and RCC risk [26].

In the present study, the pooled analysis of seven studies revealed that GC and GC+CC genotypes of rs2010963 were significantly associated with an increased risk of RCC when compared with the GG genotype. In the subgroup analysis, similar results were observed in the Asian population. If the fixed effects model was used, the C-allele of rs2010963 polymorphism was also found to increase RCC susceptibility. Besides intimate connection with the occurrence of RCC, the VEGF rs2010963 polymorphism may also be associated with more invasive biological behavior and malignancy. A previous study showed that the frequency of the CC genotype of VEGF rs2010963 polymorphism was significantly higher in advanced clinical TNM patients with a renal tumor size of more than 4 cm [15]. In advanced RCC patients receiving sunitinib therapy, the carrier C-allele of rs2010963 polymorphism is significantly related with a worse progression free survival and overall survival [27]. These findings support the hypothesis that VEGF rs2010963 polymorphism might play a role in RCC, and the C-allele might be a risk factor for RCC.

This meta-analysis also investigated the association between VEGF rs3025039 polymorphism and susceptibility to RCC. In our analysis, the results revealed that the CC genotype and CC+CT genotype were significantly associated with a decreased risk for RCC when compared with the TT genotype. However, in the subgroup analyses that excluded two studies not meeting the HWE there was no significant association. When including the studies that met the HWE status and were carried out in the Asian population, the CC+CT genotype of rs3025039 polymorphism showed a marginal significant correlation with a decreased risk of RCC when compared to those carrying the TT genotypes. In 2016, Gong et al conducted a meta-analysis and found that most genetic models and alleles displayed high susceptibility to RCC when regarding the VEGF rs3025039 polymorphism [25]. However, their study did not take in to account the heterogeneity of the included studies which were diverse in ethnicity or did not meet the HWE. We conducted a more comprehensive and cautious meta-analysis including seven eligible studies and only using random-effects models. Moreover, our subgroup analysis was based on HWE status and the Asian population. As a result we found a suspicious association which was different from the study by Gong et al. Therefore, the association between VEGF rs3025039 polymorphism and RCC needs to be validated further.

For VEGF rs699947 polymorphism, we also conducted an updated meta-analysis including six eligible studies. The pooled estimate found an increased risk for RCC in the A-carrier genotypes of VEGF rs699947 polymorphism. Our results confirmed the findings of two previous meta-analyses [25, 28]. Furthermore, subgroup analysis found that the AA genotype of rs699947 polymorphism was associated with an increased RCC susceptibility in the Asian population when compared with those carrying the CC or AC+CC genotype. Our findings indicate that the VEGF GG genotype of rs2010963 polymorphism decreased RCC susceptibility, especially in the Asian population. Moreover, the TT genotype of rs3025039 and AA genotype of rs699947 polymorphism were associated with an increased RCC susceptibility. For ethnicity variation, the possible reason may be most of our included studies were conducted in Asian and small sample size of studies conducted in Caucasian could lead to the discrepancy. When tested by sensitivity analysis, Begg's rank correlation method and Egger's weighted regression method, the results from the three SNP in VEGF did not identify any significant heterogeneity and/or publication bias.

This meta-analysis has several limitations. First, the number of studies and the sample size of the three VEGF polymorphisms were relatively small. As the individual information about the genotypes of the three VEGF polymorphisms was not sufficient, more clinical trials with larger sample size are required to address this issue. Second, only published studies were used for analysis in this study. Therefore, there might be publication bias even through tested by Begg's and Egger's method. Third, in this meta-analysis heterogeneity was detected, among the studies, which could influence the results. While the random-effects model would provide more conservative estimates with wider confidence intervals, we only used the random-effects model to calculate the pooled OR [29]. Even so, potential heterogeneity attributed by differences in ethnicity, design of the study, genotyping method, and limited sample size might not be discovered. Finally, because of insufficient individual participant data, the relationship between the haplotype of multiple VEGF polymorphisms, gene-gene and gene-environment interactions could not be estimated. If detailed information would have been available, this meta-analysis could offer more convincing evidence.

In conclusion, our meta-analysis suggests that three SNP (rs2010963, rs3025039 and rs699947) in VEGF are associated with RCC susceptibility especially in the Asian population. These SNPs in VEGF may be biomarkers for clinical evaluation. Nevertheless, additional well-designed studies including larger sample size and different ethnic groups are needed for re-evaluation of these associations.

## MATERIALS AND METHODS

### Publication search

PubMed, Institute for Scientific Information (ISI), Wangfang, Google Scholar, and Chinese National Knowledge Infrastructure (CNKI) databases were searched for all articles describing an association between the VEGF polymorphisms and RCC risk up to November 2016. The key words used were “VEGF”, “renal cancer/renal cell carcinoma”, and “polymorphism/variant”. The electronic search was supplemented by checking reference lists. All of the original studies had to meet the following criteria: (1) case-control design; (2) evaluate the association between the VEGF polymorphisms and RCC risk; (3) provide sufficient data for estimating an odds ratio (OR) with a 95% confidence interval (95% CI).

### Data extraction

Two of the authors extracted all data independently following the selection criteria. The following details were collected from each study: first author's name, publication year, country of study, ethnicity, number of cases and controls, genotyping method, and OR.

### Quality assessment

The methodological quality of each study was assessed by the Newcastle-Ottawa Scale (NOS), which uses total NOS scores (range from 0 to 9) to judge the quality of all included studies. Studies with seven or more points were considered to be of high quality.

### Statistical analysis

The OR with 95%CI was used to assess the association between the VEGF polymorphisms and RCC based on the genotype frequencies in cases and controls. For each VEGF polymorphism, the meta-analysis examined the genetic susceptibility in 5 genetic models, including allelic, homozygote, heterozygote, dominant model, and recessive model. The heterogeneity among the studies was checked by using the I^2^ value. The pooled OR was calculated for each study using only the random-effects model, considering the random-effects model is more conservative and will provide better estimates with wider confidence intervals [29]. Subgroup analyses were conducted based on the classification of the population and HWE status. Sensitivity analyses were conducted to identify the influence from single studies to the pooled estimate. Publication bias was assessed by Egger's linear regression [30] and Begg's funnel plots [31]. Quantitative synthesis analysis was performed using RevMan 5.2 software (Cochrane IMS) and STATA version 12.0 (Stata Corporation, College Station, TX, USA).

## SUPPLEMENTARY MATERIALS TABLE



## References

[1] Ferlay J, Soerjomataram I, Dikshit R, Eser S, Mathers C, Rebelo M, Parkin DM, Forman D, Bray F (2015). Cancer incidence and mortality worldwide: sources, methods and major patterns in GLOBOCAN 2012. Int J Cancer.

[2] Capitanio U, Montorsi F (2016). Renal cancer. Lancet.

[3] Hunt JD, van der Hel OL, McMillan GP, Boffetta P, Brennan P (2005). Renal cell carcinoma in relation to cigarette smoking: meta-analysis of 24 studies. Int J Cancer.

[4] Lee JE, Spiegelman D, Hunter DJ, Albanes D, Bernstein L, van den Brandt PA, Buring JE, Cho E, English DR, Freudenheim JL, Giles GG, Graham S, Horn-Ross PL (2008). Fat, protein, and meat consumption and renal cell cancer risk: a pooled analysis of 13 prospective studies. J Natl Cancer Inst.

[5] Shen T, Shu XO, Xiang YB, Li HL, Cai H, Gao YT, Zheng W, Lipworth L (2015). Association of hypertension and obesity with renal cell carcinoma risk: a report from the Shanghai Men's and Women's Health Studies. Cancer Causes Control.

[6] Roskoski R (2017). Vascular endothelial growth factor (VEGF) and VEGF receptor inhibitors in the treatment of renal cell carcinomas. Pharmacol Res.

[7] Vincenti V, Cassano C, Rocchi M, Persico G (1996). Assignment of the vascular endothelial growth factor gene to human chromosome 6p21.3. Circulation.

[8] Kim KJ, Li B, Winer J, Armanini M, Gillett N, Phillips HS, Ferrara N (1993). Inhibition of vascular endothelial growth factor-induced angiogenesis suppresses tumour growth in vivo. Nature.

[9] Ferrara N (1993). Vascular endothelial growth factor. Trends Cardiovasc Med.

[10] Kaya A, Ciledag A, Gulbay BE, Poyraz BM, Celik G, Sen E, Savas H, Savas I (2004). The prognostic significance of vascular endothelial growth factor levels in sera of non-small cell lung cancer patients. Respir Med.

[11] Toulis KA, Goulis DG, Mintziori G, Kintiraki E, Eukarpidis E, Mouratoglou SA, Pavlaki A, Stergianos S, Poulasouchidou M, Tzellos TG, Makedos A, Chourdakis M, Tarlatzis BC (2011). Meta-analysis of cardiovascular disease risk markers in women with polycystic ovary syndrome. Hum Reprod Update.

[12] Iordache S, Saftoiu A, Georgescu CV, Ramboiu S, Gheonea DI, Filip M, Schenker M, Ciurea T (2010). Vascular endothelial growth factor expression and microvessel density--two useful tools for the assessment of prognosis and survival in gastric cancer patients. J Gastrointestin Liver Dis.

[13] Ajaz S, Khaliq S, Abid A, Hassan AS, Hashmi A, Sultan G, Mohsin R, Mubarrak M, Naqvi SA, Rizvi SA, Mehdi SQ (2011). Association of a single-nucleotide polymorphism in the promoter region of the VEGF gene with the risk of renal cell carcinoma. Genet Test Mol Biomarkers.

[14] Ma N, Li LW, Cheng JL (2015). Predictive value of vascular endothelial growth factor polymorphisms on the clinical outcome of renal cell carcinoma patients. Oncol Lett.

[15] Zhong W, Wang X, Pan B, Su Z (2014). Association of vascular endothelial growth factor polymorphisms with clinical outcome of renal cell carcinoma patients. Tumour Biol.

[16] Qin C, Chen J, Li J, Ju X, Zhang S, Cao Q, Han Z, Li P, Shao P, Wang M, Zhang Z, Gu M, Zhang W (2014). Variants in angiogenesis-related genes and the risk of clear cell renal cell carcinoma. Mutagenesis.

[17] Shen BL, Qu QS, Miao SZ, Zhang YX (2015). Association between SNPs in vascular endothelial growth factor polymorphisms and risk of renal cell carcinoma: a case-control study. Genet Mol Res.

[18] Saenz-Lopez P, Vazquez F, Cozar JM, Carretero R, Garrido F, Ruiz-Cabello F (2013). VEGF polymorphisms are not associated with an increased risk of developing renal cell carcinoma in Spanish population. Hum Immunol.

[19] Bruyere F, Hovens CM, Marson MN, d'Arcier BF, Costello AJ, Watier H, Linassier C, Ohresser M (2010). VEGF polymorphisms are associated with an increasing risk of developing renal cell carcinoma. J Urol.

[20] Kawai Y, Sakano S, Korenaga Y, Eguchi S, Naito K (2007). Associations of single nucleotide polymorphisms in the vascular endothelial growth factor gene with the characteristics and prognosis of renal cell carcinomas. Eur Urol.

[21] Lu G, Dong Y, Zhang Q, Jiao L, Yang S, Shen B (2015). Predictive value of vascular endothelial growth factor polymorphisms on the risk of renal cell carcinomas: a case-control study. Tumour Biol.

[22] Kushner EJ, Bautch VL (2013). Building blood vessels in development and disease. Curr Opin Hematol.

[23] Rahoui J, Laraqui A, Sbitti Y, Touil N, Ibrahimi A, Ghrab B, Al Bouzidi A, Moussaoui Rahali D, Dehayni M, Ichou M, Zaoui F, Mrani S (2014). Investigating the association of vascular endothelial growth factor polymorphisms with breast cancer: a Moroccan case-control study. Med Oncol.

[24] Maltese P, Canestrari E, Ruzzo A, Graziano F, Falcone A, Loupakis F, Tonini G, Santini D, Magnani M (2009). VEGF gene polymorphisms and susceptibility to colorectal cancer disease in Italian population. Int J Colorectal Dis.

[25] Gong M, Dong W, Shi Z, Qiu S, Yuan R (2017). Vascular endothelial growth factor gene polymorphisms and the risk of renal cell carcinoma: evidence from eight case-control studies. Oncotarget.

[26] Yang SM, Huang CY, Shiue HS, Huang SP, Pu YS, Chen WJ, Lin YC, Hsueh YM (2015). Joint effect of urinary total arsenic level and VEGF-A genetic polymorphisms on the recurrence of renal cell carcinoma. PLoS One.

[27] Kim JJ, Vaziri SA, Rini BI, Elson P, Garcia JA, Wirka R, Dreicer R, Ganapathi MK, Ganapathi R (2012). Association of VEGF and VEGFR2 single nucleotide polymorphisms with hypertension and clinical outcome in metastatic clear cell renal cell carcinoma patients treated with sunitinib. Cancer.

[28] Aji Kaisaier, M M, Rexiati Mulati, Bai Yong, Cui Lei (2016). Association between vascular growth factor gene −2578C>A, +1612G>A polymorphism and the risk of renal cell carcinoma: a meta-analysis. Int J Clin Exp Med.

[29] Kontopantelis E, Reeves D (2012). Performance of statistical methods for meta-analysis when true study effects are non-normally distributed: a simulation study. Stat Methods Med Res.

[30] Egger M, Davey Smith G, Schneider M, Minder C (1997). Bias in meta-analysis detected by a simple, graphical test. BMJ.

[31] Begg CB, Mazumdar M (1994). Operating characteristics of a rank correlation test for publication bias. Biometrics.

