# Supplementary material for: Association between three VEGF polymorphisms and renal cell carcinoma susceptibility: a meta-analysis
Source: Oncotarget. 2017 May 12;8(30):50061–70. doi: 10.18632/oncotarget.17833 (PMC5564828; doi:10.18632/oncotarget.17833)
Supplement: Supplementary file 1 [file oncotarget-08-50061-s001.pdf]

Association between three VEGF polymorphisms and renal cell carcinoma susceptibility: a meta-analysis

SUPPLEMENTARY TABLE

Supplementary Table 1: Quality assessment based on the Newcastle-Ottawa Scale of studies included in this meta-analysis

Author	Year	Adequate definition of case	Representativeness of cases	Selection of control	Definition of control	Control for important factor or additional factor	Exposure assessment	Same method of ascertainment for cases and controls	Nonresponse rate	Total quality scores
Abe	2002	★	★		★	★	★	★		6
Bruyère	2010	★	★	★	★	★	★	★		7
Ajaz	2011	★	★		★	★★	★	★		7
Sáenz-López	2013	★	★	★	★	★	★	★		7
Chao	2014	★			★	★★	★	★		6
Lu	2015	★	★		★	★★	★	★		7
Xian	2015	★	★		★	★★	★	★		7
Shen	2015	★	★		★	★★	★	★		7
Yang	2015	★	★		★	★★	★	★		7
